# Repeat infections with chlamydia in women may be more transcriptionally active with lower responses from some immune genes

**DOI:** 10.3389/fpubh.2022.1012835

**Published:** 2022-10-10

**Authors:** Wilhelmina M. Huston, Amba Lawrence, Bryan A. Wee, Mark Thomas, Peter Timms, Lenka A. Vodstrcil, Anna McNulty, Ruthy McIvor, Karen Worthington, Basil Donovan, Samuel Phillips, Marcus Y. Chen, Christopher K. Fairley, Jane S. Hocking

**Affiliations:** ^1^Faculty of Science, University of Technology Sydney, Ultimo, NSW, Australia; ^2^Institute of Health and Biomedical Innovation, Queensland University of Technology, Kelvin Grove, QLD, Australia; ^3^Bioinnovation Centre, University of the Sunshine Coast, Sippy Downs, QLD, Australia; ^4^Melbourne Sexual Health Centre, Central Clinical School, Monash University, Carlton, VIC, Australia; ^5^Centre for Epidemiology and Biostatistics, Melbourne School of Population and Global Health, University of Melbourne, Carlton, VIC, Australia; ^6^Sydney Sexual Health Centre, Sydney, NSW, Australia; ^7^Melbourne Sexual Health Centre, Alfred Health, Carlton, VIC, Australia; ^8^Kirby Institute, University of New South Wales, Kensington, NSW, Australia; ^9^Melbourne School of Population and Global Health, University of Melbourne, Carlton, VIC, Australia; ^10^Australia and Melbourne Sexual Health Centre, Carlton, VIC, Australia

**Keywords:** *Chlamydia*, sexually transmitted infection, repeat infections, genomics, gene expression, azithromycin, antibiotic

## Abstract

*Chlamydia trachomatis*, the most common bacterial sexually transmitted infection worldwide, is responsible for considerable health burden due to its significant sequelae. There are growing concerns about chlamydial treatment and management due to widely documented increasing burden of repeat infections. In the current study, a cohort study design of 305 women with urogenital chlamydial infections demonstrated that 11.8% of women experienced repeat infections after treatment with azithromycin. The chlamydial DNA load measured by quantitative PCR was higher in women who experienced a repeat infection (*p* = 0.0097) and repeat infection was associated with sexual contact. There was no genomic or phenotypic evidence of azithromycin resistance within the chlamydial isolates. During repeat infection, or repeat positive tests during follow up, vaginal chlamydial gene expression (*ompA, euo, omcB, htrA, trpAB*) was markedly higher compared to baseline, and two of the selected immune genes analyzed had significantly lower expression at the time of repeat infection. Overall, there are two implications of these results. The results could be generalized to all recent infections, or repeat positive events, and indicate that chlamydial infections are have higher transcriptional activity of select genes early in the infection in women. Alternatively, after azithromycin treatment, repeat infections of *Chlamydia* may be more transcriptionally active at certain genes, and there may be post-treatment immunological alterations that interplay into repeat exposures establishing an active infection. The potential that recent infections may involve a higher level of activity from the organism may have implications for management by more regular testing of the most at risk women to reduce the risk of sequelae.

## Introduction

*Chlamydia trachomatis* continues to be the most commonly diagnosed bacterial sexually transmitted infection (STI) globally (1). It can have adverse health consequences particularly for women [reviewed (2)] with an estimated 17% of cases leading to pelvic inflammatory disease (3) and an estimated 45% of tubal factor infertility being attributable to past *Chlamydia* infection (4). Several studies have reported high *Chlamydia* repeat infection rates among young women re-tested following treatment. An Australian cohort of 1,116 young women found that among those women testing positive at baseline, 18% tested positive again at 3 months following treatment (5). Other studies have reported similarly high rates of repeat infection, ranging from 21% within 6 months in New Zealand (6) to 29.9% per year after treatment in the UK and 34% per year (7) in the USA.

Repeat infections may represent: (1) re-infection due to unprotected sexual contact with an infected partner; (2) treatment failure as a result of noncompliance with treatment, poor absorption of the drug, reduced antimicrobial susceptibility, or antimicrobial resistance; (3) persistence due to host or microbial factors such as immune response or other undefined host factors; or (4) auto-inoculation from a persistent rectal chlamydia infection that has not been effectively treated (8–12). There is increasing concern that treatment failure or auto-inoculation from a persistent rectal infection may account a significant proportion of repeat urogenital infections in women (13, 14). Rectal chlamydia is detected in about 80% of women who are diagnosed with urogenital infection (15). Azithromycin is still widely used to treat urogenital infection but is substantially less effective at clearing rectal chlamydia (16), increasing the risk that any concurrent rectal infection could subsequently auto-inoculate causing a repeat urogenital infection. There have not been any confirmed azithromycin resistant isolates, although there are some conflicting reports (17, 18), and there is laboratory evidence that such isolates are unfit and unlikely to be maintained in the population (19). A partner treatment study found that among female participants who reported no sexual intercourse after treatment, 22 of 289 (8%; 95% CI: 5–11%) had persistent infection at follow up, suggestive of treatment failure (20). A cohort of adolescent females also found a treatment failure rate of 7.9% (95% CI: 4–10.1%) (7). There is also *in vitro* evidence that *Chlamydia* can enter a persistent form where infected cells exposed to β-lactam antibiotics, interferon-γ or deprived of iron or amino acids, can exhibit persistence (8, 9, 11, 21, 22). This allows chlamydia to remain dormant, non-infectious and undetectable by culture but, on removing the stressful conditions, it can be recovered by culture. There is also evidence that latent infection may not be detectable, even using PCR, if only cells shed from the mucosal surface are sampled (22–24). It is not known how often this persistent state occurs *in vivo* and whether removal of treatment can trigger reactivation.

We conducted a cohort study of women diagnosed with urogenital chlamydia to investigate factors associated with repeat infection in women treated with azithromycin, including examining human and microbial factors. Here we report genotypic and phenotypic analysis of the chlamydial isolates and some host responses from this cohort study.

## Materials and methods

### Cohort design and analysis

This study was a cohort study to examine repeat infections in women with urogenital chlamydial infections treated with Azithromycin, the Australian Chlamydia Treatment Study (25). In brief 305 women aged ≥ 16 diagnosed with NAAT positive genital chlamydia were recruited from two large sexual health clinics in Sydney and Melbourne Australia between October 2012 and October 2014. Women were eligible for inclusion if they had adequate English language skills to give consent and remained in the local area for 8 weeks. Women were recruited when they returned to the clinic for treatment for their initial chlamydia infection. They completed a survey and provided four clinician collected high vaginal swabs for testing. Women were excluded if they had a concomitant STI, had concurrent PID, were commercial sex workers, had taken another antibiotic within the last 2 weeks, did not have a mobile phone or an address to which parcels could be posted, were HIV positive or had a macrolide allergy or were taking other medications likely to interact with azithromycin. The proportion and 95% confidence intervals of those who had a repeat infection was calculated using exact binomial methods accounting for clustering at the clinic level. The incidence or repeat infection and 95% confidence intervals were calculated using poisson methods. Cox proportional hazards regression was used to calculate factors associated with time till repeat infection. Factors investigated included socio-demographic and behavioral variables. Given relatively small number of cases of repeat chlamydia, only unadjusted Cox regression was performed.

Associations between repeat positive testing events and chlamydia organism at the index by IFU or PCR load were investigated for all those participants who had a test of cure at week 4 using *t*-tests where measures of load were log transformed. It was not always possible to measure load for all available baseline samples due to factors such as heavy contaminants of blood or other organisms interfering in assays, low chlamydial load, or poor swab collection quality (see Supplementary Table S1).

All repeat positive index and follow up samples were cultured where possible and of these, 19 samples from index eight samples from follow up were able to be purified from other contaminants and included in the minimum inhibition concentration (MIC) measures. A further 26 index samples from women who were negative on follow up (follow up negative, FoN) were also able to be cultured and purified from other contaminants and included in the MIC analysis. These isolates represented 26 samples at indexfrom women with no repeat positives (FoN), 19 samples from index from women who experienced a FoP and eight samples at a test of cure (4 weeks), from women who experienced positive follow up results (FoP).

MIC (minimum inhibitory concentration) was conducted on chlamydial cultures in McCoy B cell monolayers following the protocol previously described (12). Azithromycin was added to the cultures in a twofold dilution dose series (μg/ml), cultures conducted, fixed and labeled using immunocytochemistry to examine chlamydial inclusions using our in-house method [described (26, 27)]. MIC_tp_ or transition point MIC, was defined as the dose at which 90% or more of the inclusions were altered in size and morphology. The presence of 10% or less visible standard inclusions was used to determine the MIC_tp_, with the MIC considered to be the next higher dose in the series from the MIC_tp_. Resistance is considered to be a MIC of 4 μg/ml or greater for *Chlamydia* (12). The MIC of cultured isolates to azithromycin at baseline were calculated and compared between those with and without repeat chlamydia infection using *t* tests.

### Gene expression analysis

A nested Case-Control study was conducted within the cohort study where a group of women from the study (12 case- Follow up Positive, control- Follow up Negative) were selected based on matching for age and contraceptive usage (two controls per case) and included in the gene expression analysis. See Supplementary materials for further characteristics of these cases and controls (Supplementary Table S2). Total RNA was then extracted and purified from each sample using the Purelink™ RNA Mini Kit (Thermo Fisher Scientific), in accordance with the manufacturer's instructions. The concentration and purity of each RNA sample was assessed using the NanoDrop™ One/One C UV-Vis spectrophotometer (Thermo Fisher Scientific). Complementary DNA (cDNA) synthesis with random hexamer priming was conducted using the SuperScript™ III First Strand Synthesis Reverse Transcription kit (Thermo Fisher Scientific). The relative human gene expression levels in cDNA samples were analyzed using reverse-transcription quantitative polymerase chain reaction (RT-qPCR). The genes of interest included in the analysis were IDO1, IRF-1, FTH-1, IL-6, IL-8, IL-1α, TNF-α, IL10, IFN-γ. The expression levels of each human gene of interest was determined relative to the geometric mean of glyceraldehyde-3-phosphate dehydrogenase (*gapdh*) and phosphoglycerate kinase 1 (*pgk1*) cDNA levels. Chlamydial genes were analyzed by normalization against 16srRNA DNA levels (as an indicator of chromosome counts). Primers are provided in Supplementary Table S3. The oligonucleotide sequences used to amplify and measure chlamydial *16S rRNA* (28)*, euo* (29) and *ompA* (30) cDNA were from previously published studies, while those used to amplify *htrA, trpBA* and *omcB* cDNA were designed using the NCBI Primer Blast tool. The genome of *C. trachomatis* type strain D/UW-3/Cx (accession number NC_000117) was used as the template for primer design; however, primers were only selected if they amplified from control cultures of in-house stocks of all genovars in the present study.

Each of the primers used were validated by conventional PCR and agarose gel electrophoresis, before the efficiency of each primer set was determined. Only primers with an efficiency of between 90 and 110% were used during this study. The mean cycle threshold (Ct) value of each of the participant cDNA samples was calculated from technical replicates, which were repeated or excluded if the mean Ct value had a standard deviation greater than 1.0.

Raw RT-qPCR data was exported from the Rotor-Gene Q Series software platform and stored and transformed using Microsoft Excel. For each sample, the mean Ct value of each gene of interest was subtracted from the geometric mean of *gapdh* and *pgk1*, to obtain the delta cycle threshold (ΔCt) value. These ΔCt values were then transformed using means and standard deviation for each gene to ΔΔCt using negative log transformation. The chlamydia genes were first normalized to the quantity of chromosomal DNA of the 16S rRNA chromosome for that sample before transformed using means and standard deviation for each gene to ΔΔCt using negative log transformation. The analysis was Baseline: No Repeat – Baseline: Repeat infection, and Baseline: Repeat Infection – Repeat Infection. The transformed ΔΔCt datasets were graphed in Graphpad Prism 7. Statistical comparisons were conducted using Graphpad Prism 7, with the Mann-Whitney U used test to determine whether any significant differences between two groups using the non-transformed ΔΔCt datasets. Paired sample analysis was conducted using the Wilcoxon test prior to log transformed ΔΔCt for graphical display.

### Genomic analysis

Genome sequences of the *Chlamydia* were examined using two approaches. One approach was whole genome sequencing of cultured isolates on a small selection of the specimens collected. The cases of repeat infection where chlamydial isolates were able to be cultured were included in this genomic analysis, were selected to ensure representation across a variety of *ompA* genotypes, and able to be cultured and selected away from contaminants during the culture. This included 15 isolates (from 11 participants); eight were the index and FoP events from four participants, four were associated with FoP but from index isolates only, and 3 from index swabs from women who did not experience repeat positive (FoN) (Supplementary Table S1).

The second approach, using sequence capture and enrichment, was attempted on index and follow up positive samples from all participants who experienced a follow up positive test. The aim was to sequence and analyse *chlamydial* DNA directly from high vaginal swabs from both events in each participant to characterize any genomic features that might associate with repeat infection. All swabs from index and FoP event in the same participant were included in the sequence capture genomics methods, although several failed to recover adequate DNA or sequencing read depth for genomic analysis and were excluded from further analysis (Supplementary Table S1).

The baseline and repeat infection swabs collected in SPG (chlamydial storage solution) were used to extract DNA for this analysis. The reads were filtered with a Q20 threshold for quality, and stringent read filtering thresholds were used in bowtie2 (31) and samtools (32) to remove reads from other bacteria, and to allow mapping of reads against a reference from *C. trachomatis* D/UW. A selection of 15 isolates were cultured and sequenced from DNA extractions from the cultures (as many as 12 culture passages from the primary swab were used for these extractions). The DNA extracts were sequenced using an Illumina MiSeq using the TruSeq v3 reagents generating 300 bp long paired-end reads. The sequence library was prepared using and Illumina NexteraXT Library Preparation Kit. Paired end reads were quality checked for consistency in base qualities using FastQC v0.11.2 and were trimmed using Trimmomatic v0.32 (33). Reads were analyzed using a sliding window of 4 bp and trimmed once the average base quality across the window dropped below 28. A minimum read length of 50 bps was required after trimming. Only bases 5 to 200 of each read was retained due to the lower average quality of the bases outside this range. Reads were mapped to a reference published *C. trachomatis* strain D/UW/3-CX genome using both Shrimp v2.2.3 (34) and Bowtie v.2.2.3 (31) with only the best match reported for each read. SNPs and indels were filtered, called and annotated using the Nesoni data analysis toolset (Harrison, unpublished). Reads were assembled with Velvet v1.2.10 (35). Optimal *k-*mer length for De Brujin graph construction was determined using the VelvetOptimiser v2.2.5 wrapper script (Gladman & Seeman, unpublished). Assemblies were optimized using PAGIT to close gaps by comparing against a reference genome (36). Assembled genomes were annotated using Prokka v1.10 (37).

A phylogeny was constructed using kSNP v2.1.2 (38) with a *k-*mer of 17, as estimated by Kchooser to the relationships of the unassembled isolates relative to 62 other *C. trachomatis* strains. The genome sequences of these strains were retrieved from the NCBI RefSeq database in October 2014. A whole genome alignment was performed using Mugsy v1.2.3 (39) using the default parameters and a Maximum-Likelihood phylogeny estimated using RAxML (v8.1.15) using the rapid hill-climbing algorithm (-f a) and the GTR model of nucleotide substitution with a gamma distribution of rate variation among sites (-m GTRGAMMA). The resulting alignment contained 5,854 phylogenetically informative sites.

### Biospecimen handling

Biospecimen collection was conducted as previously outlined (25), unless otherwise described here. Diagnostic PCR, genovar profiling PCR (40), and *Chlamydia* culture and enumeration (41) were all conducted in accordance with previous publications. These samples were selected firstly be identifying a variety of cases that were associated with distinct chlamydial *ompA* genomes to get representation across the range of pathogens that could then be matched to two controls by age and contraceptive type in usage. Chlamydial genome sequences were analyzed either directly from the participant swabs or from isolates cultured from the swabs as outlined in the genomic analysis.

## Results

### Chlamydial burden in the absence of *in vitro* resistance, was associated with repeat infections

A total of 305 women were enrolled into the cohort; 271 (88.9%) had a test of cure at week 4, and 223 (73.1%) were followed until study end at week 8. The median time for follow up was 56 days (IQR: 44–58 days), with a total of 2,154 weeks of follow up. Overall, 36 (11.8%; 95%CI: 9.4, 14.7) had a repeat infection during follow up, with an incidence of 1.7 repeat infections per 100 weeks (95%CI: 1.6, 1.8). Time to repeat positive tests during follow up was associated with 2 or more partners in the last week compared with no partners (HR = 3.4; 95%CI:1.1, 10.4) (Table 1). No other socio-behavioral variables were significantly associated with repeat infection.

**Table 1:** Factors associated with positive tests on follow up among women in the cohort.

**Variable**	No. repeat infections*	**Person time (weeks)**	**Unadjusted HR (95%CI)**
**Age**			
16–20	8	355.1	1.0
21–25	18	1,182.9	0.6 (0.3, 1.5)
26–30	6	436.6	0.5 (0.2, 1.6)
30+	4	179.9	0.7 (0.2, 2.4)
**Country of birth**			
Other	22	1,402.4	1.0
Australia	13	747.9	1.2 (0.6, 2.4)
**Use of hormonal contraception**
No	10	689.7	1.0
Yes	26	1,464.7	1.4 (0.7, 3.0)
**Use of condoms as contraception**
No	14	764.1	1.0
Yes	22	1,390.3	0.8 (0.4, 1.6)
**Number of partners 12 month before study**
1 partner	2	152.3	1.0
2–4 partners	14	876.6	1.1 (0.2, 4.7)
5–9 partners	13	877.1	0.9 (0.2, 4.1)
10+	7	248.4	2.1 (0.4, 10.2)
**Number of partners in last week**
0	13	1,131.4	1.0
1	19	937.7	1.4 (0.7, 2.8)
2 or more	4	85.3	3.4 (1.1, 10.4)
**At least one episode of anal sex last week**
No	34	2,101.9	1.0
Yes	2	52.6	3.4 (0.8, 14.3)
**Condom used with all vaginal sex partners last week**
No	16	704.1	1.0
Yes	8	347.1	1.3 (0.5, 2.7)
No sex	12	1,103.1	0.6 (0.3, 1.3)

*Some missing data so numbers will not always add up to 36 cases.

**Figure 1:**
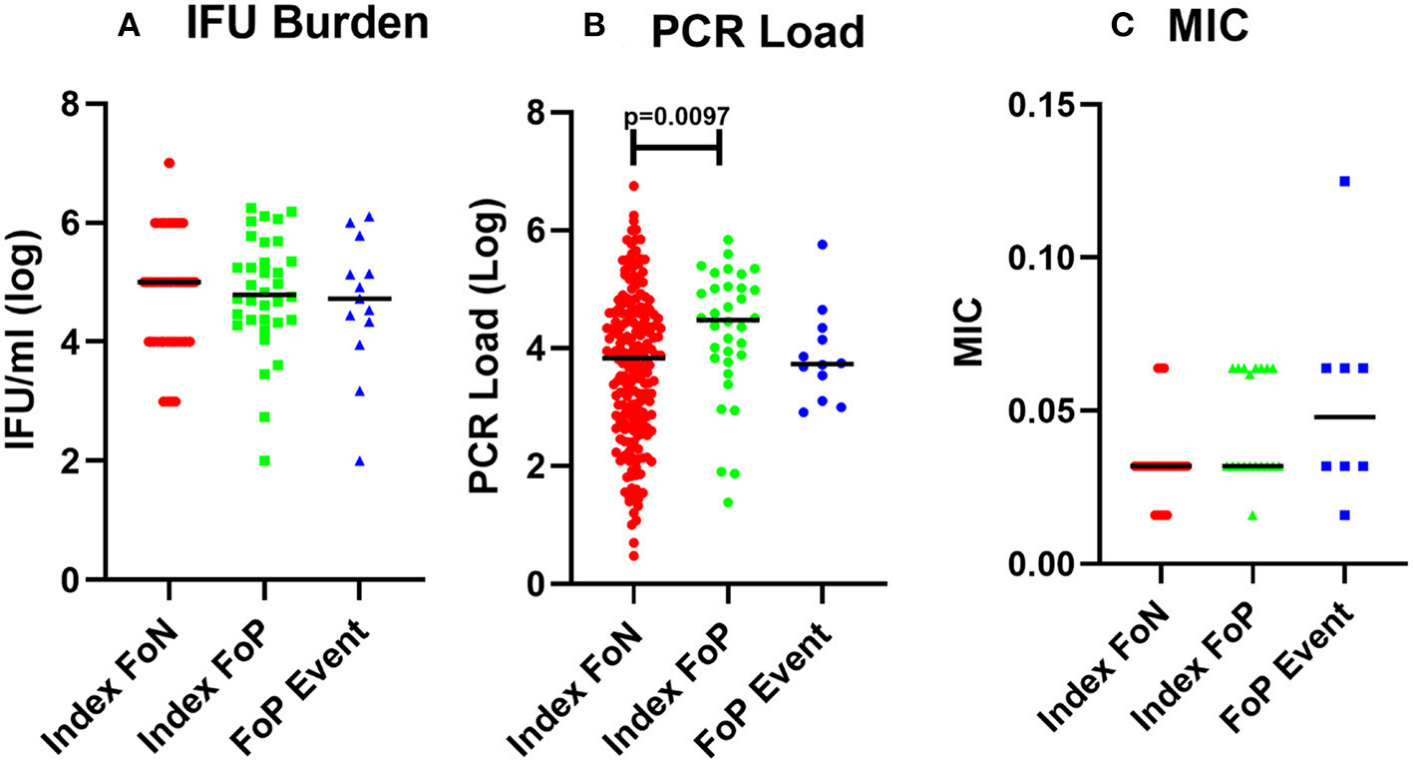
Chlamydial burden and Minimum inhibitory concentration of azithromycin against isolates from participants who experienced a positive test during follow up. **(A)** cultured inclusion forming units per ml from high vaginal swabs (log), participant data included includes Index FoN (*n* = 235), Index FoP (*n* = 36), FoP Event (*n* = 31). **(B)** Quantitative PCR assay for CT organism load (log). Data includes participant results from; Index FoN (*n* = 224); Index FoP (*n* = 34); and FoP Event (*n* = 36). **(C)** MIC from cultured chlamydia isolates. The data include the MIC for isolates from participants in the following groups; Index FoN (*n* = 26 isolates); Index FoP isolates (*n* = 19); and FoP Event isolates (*n* = 8). Data are shown by these groups as indicated on the x axis. The measures are shown on the y-axis. Summary data are also provided in Table 1.

Analysis of organism burden by molecular assay identified a higher load of *Chlamydia* in women at index who experienced positive test during follow up (FoP) (Figures 1A,B, Table 2). MIC of azithromycin against the chlamydial isolates was determined to be within the susceptible range for all isolates that were cultured (*n* = 53) (see Materials and methods, results see Table 2). One isolate showed an elevated MIC (0.125 μg/ml) when cultured from repeat infection compared to the baseline culture but remained in the range considered susceptible (Figure 1C, Table 2). All isolates were susceptible to azithromycin.

**Table 2:** Microbial features associated with positive follow up test.

	**Index FoN**	**Index FoP**	***p* value (Index FoP – Index FoN) f^a^**	**FoP Event**	***p* value (Index FoP vs. FoP event)**
**IFU/ml Raw data**					
Mean	1, 918 53.1	280, 511.8	*index expression of any of the selected* = 0.7168	109, 079.4	*p* = 0.6561
SD	496, 048.7	471, 819.4		297, 597	
Median	10, 858	55, 104.35		0	
IQR	0–120, 342.8	19, 517.25–221, 548.4		0- 33, 659.8	
Range	0–3, 474 560	0–1, 769 854		0- 1, 262 243	
n	235	36		31	
**Organism Burden by DNA detection (PCR load)**					
Mean	9, 2456.06	86, 024.57	*p =* 0.0097	18, 937.44	*p =* 0.5968
SD	424, 404.1	139, 072.3		95, 166.28	
Median	4,330	28, 000		0	
IQR	339.5–31, 650	5, 780–104, 200		0-2910.53	
Range	0- 5, 620 000	0–684, 000		0–571, 900	
n	224	35		36	
**MIC of cultured isolates to Azithromycin** ^**b**^					
Mean	0.033	0.044	*p =* 0.0216	0.054	*p =* 0.7198
SD	0.013	0.017		0.034	
Min-Max	0.016–0.064	0.016–0.064		0.016–0.125	
n	26	19		8	

^**a**^Compared using t-test, ^b^compared using Mann-Whitney U test.

Note statistical analysis was conducted on log transformed data as shown graphically in Figure 1.

*Some missing data.

### Chlamydial repeat infection isolates do not have genomic resistance or a unique genotype

Whole genome sequencing and phylogenetic analysis of *Chlamydia* genomes from 28 women included in the analysis show that the *C. trachomatis* identified in these specimens did not belong to a specific lineage and represented a phylogenetically heterogeneous population likely reflective of the organism's global transmission.

Index and follow up positive swabs were successfully sequenced and analyzed from 25 participants, a total of 50 paired samples, using the sequence capture approach. In the case of six participants only one swab was able to be sequenced and analyzed using the sequence capture approach. This provided genomic insights into 25 individuals who experienced repeat infections (Supplementary Table S4).

**Figure 2:**
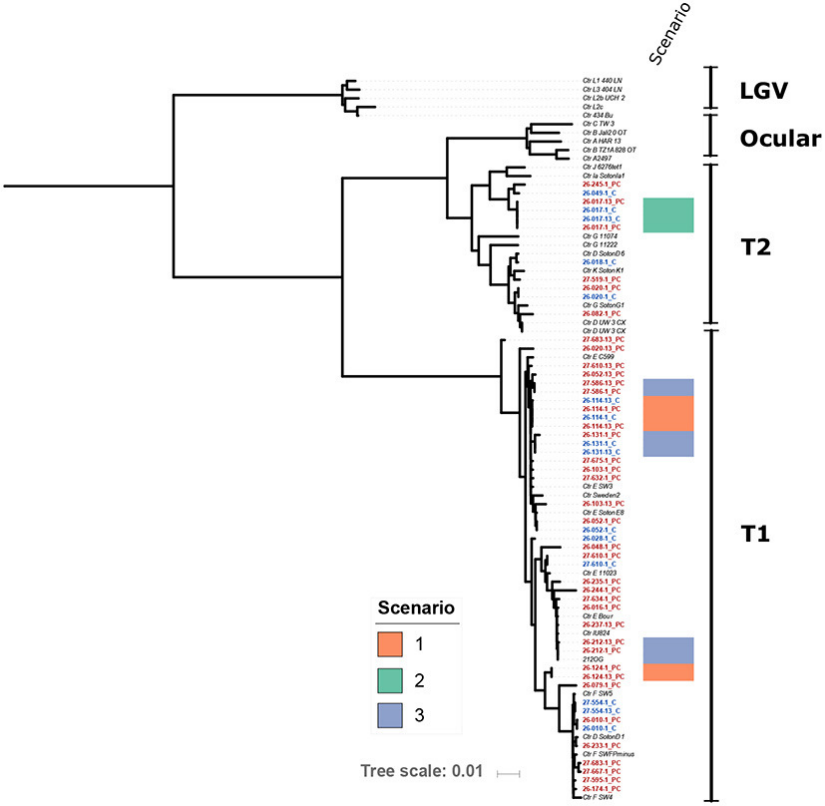
Maximum-Likelihood core genome phylogeny of the study isolates with 29 reference *Chlamydia trachomatis* (Ctr) genomes (*italicized*) based on 5,854 phylogenetically informative core SNPs. Isolates from this study are highlighted with red and blue font; red font indicates probe capture (PC) isolates and blue indicates cultured genomes. The position of isolates from Scenarios 1, 2, and 3 are indicated as shown by the color key. Study isolates that do not have a scenario indicated on the figure are those from Scenario 4 as described in the discussion, or where there was not enough resolution in one swab to define the Scenario. Scenario 1 is defined as identical isolate genotypes and the participant reported either/or both sexual contact with a repeat sexual partner and a history of anal sex. Scenario 2 is defined as identical isolate genotypes and the participant reported no sexual contact and no history of anal sex. Scenario 3 is defined as low genetic differences, and participants reported a variety of sexual behaviors from no sexual contact to sexual contact with a repeat partner and a history of anal sex. Scenario 4 indicates where the genomics supports a completely different isolate, in these scenarios participants reported a variety of sexual behaviors from no sexual contact to sexual contact with a repeat partner and a history of anal sex.

The genomics datasets were analyzed from both approaches using phylogeny analysis of the whole genome sequence and there was no clustering of chlamydial genomes associated with repeat infections (Figure 2). The sequences of genetic loci previously associated with resistance to azithromycin, 23s rRNA (ribosomal structural gene and target site of azithromycin), L4 (a ribosomal associated with SNPS identified in azithromycin resistance), and L22 (a ribosomal associated with SNPS identified in association with azithromycin resistance), were examined and no polymorphisms corresponding to known resistance mechanisms previously described in other organisms were present, regardless of if they were associated with follow up positive event or not.

Analysis of the genomics of 25 paired isolates from the women who experienced a positive test result during follow up (Table 3, Figure 2), was conducted in conjunction with the datasets reported by each individual for sexual behavior. The follow up positive sequence capture analysis had sometimes lower read depths compared to baseline (Supplementary Table S4). In three cases identical chlamydial genomic sequences were detected for the baseline and repeat infection events. Two of these cases reported exposure to a repeat sexual partner and a recent history of/or current anal sex. One of these cases reported no current or recent history of anal sex and no sexual contact during the study period. There were three cases with a low number of genomic variations between the index and positive follow up event, with a variety of sexual behavior from no activities to repeated sexual activities with a recent partner reported. 19 cases had clearly different isolates present in the index and follow up positive events with 301 or greater genomic distinctions. In these cases, where the genomics clearly indicated the presence of a genetically distinct chlamydial isolate from the isolate at baseline, there were some participants who reported no sexual exposure, and no recent history of anal sex.

**Table 3:** Genomics analysis of positive follow up events in the context of sexual behavior.

**Genomic**	**Number of**			**Scenario (outlined**
**Differences (range)**	**participants**	**Read depth**	**Sexual behavior^!^**	**in discussion)**
0	2	Mean = 1,191.5	Repeat sexual exposure, recent anal sex	1
		SD = 409.6		
		Range = 718–1,566		
		*n =* 4		
0	1	Mean = 1528.5	No repeat sexual exposure, no recent anal sex	2
		SD = 95.5		
		Range = 161–1,596		
		*n* = 2		
24–94	3	Mean = 710.8	Repeat sexual exposure, recent anal sex reported	3
		SD = 628.1		
		Range = 54–604		
		*n =* 6		
301–4,725	19	Mean = 651	Repeat sexual exposure/reported no repeat sexual exposure, recent anal sex/no anal sex	4
		SD = 593		
		Range 8–1,570		
		*n =* 38		

^!^Repeat exposure is defined as sexual contact with a repeat sexual partner during study time frame, recent anal sex includes in recent past or during study timeframe.

The genomics data and these scenarios was used to further consider the role of infectious burden at the index time point in the outcome of the follow up positive test events. Among women who were likely to have had the same infection, chlamydial DNA load at time of index case was significantly higher than for those women who did not have a positive test during follow up (*p* = 0.0338; mean 3.65 vs. 4.72 log10). In contrast, there was less evidence of a difference in chlamydial DNA load at time of index case between those women who had positive or negative follow up results (0.0781; mean 3.65 versus 4.19 log10).

### Chlamydial genes were more highly expressed in the repeat infection event, and some immune genes were down regulated

**Figure 3:**
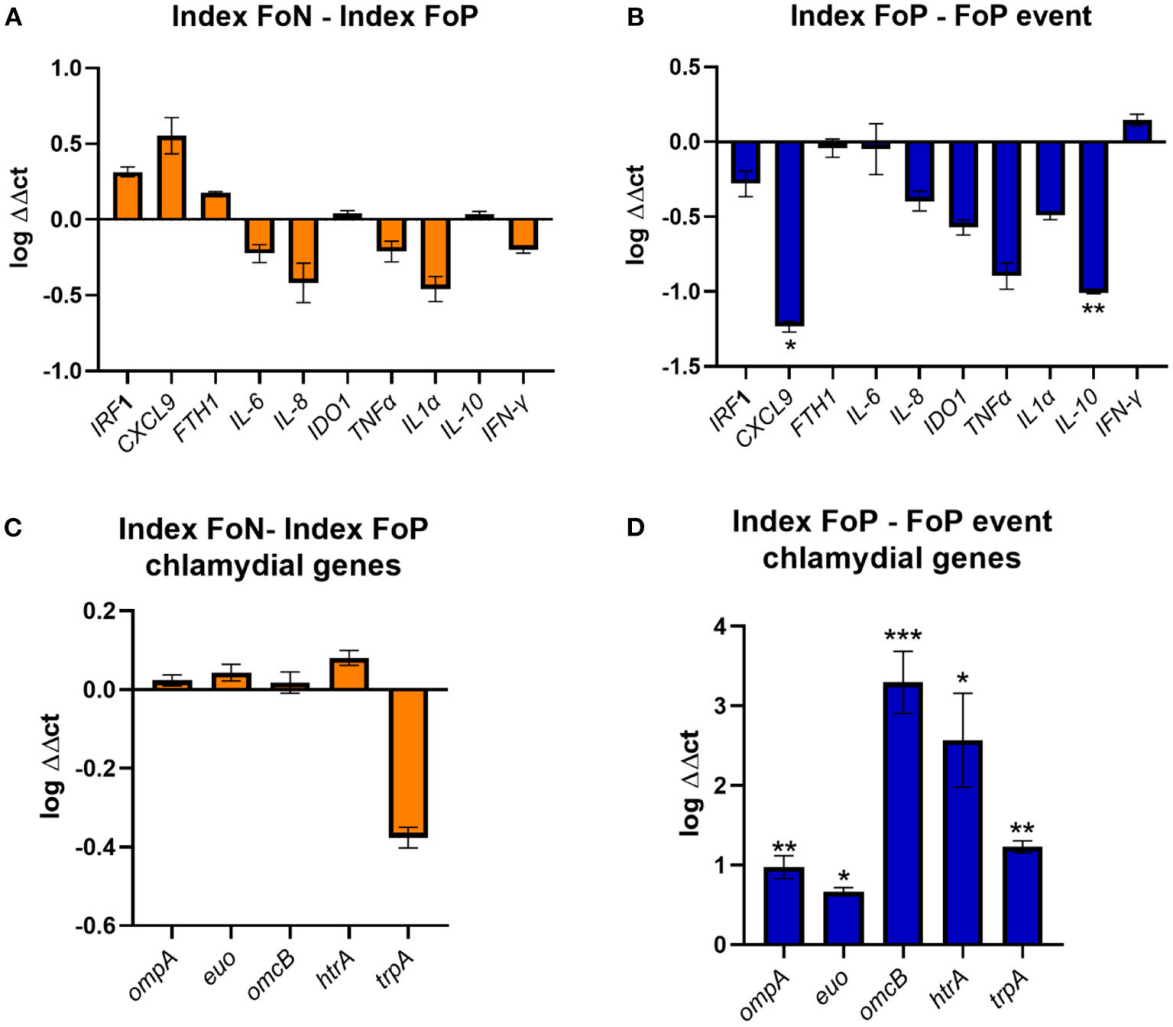
Expression of immune genes from vaginal swabs. **(A)** The figure shows the vaginal immune gene expression shown as ΔΔct log transformed data comparing the Index Follow up Negative (Index FoN) to Index Follow up positive (Index FoP) (orange) for the human genes analyzed. **(B)** The graph shows the vaginal immune gene expression shown as ΔΔct log transformed data comparing the Index FoP with the FoP event in the same participants for the human genes analyzed. **(C)** The figure shows the vaginal immune gene expression shown as ΔΔct log transformed data *C. trachomatis ompA, euo, omcB, htrA*, and *trpBA* normalized to *Ct* 16S rRNADNA copies as a normaliser for the comparison of the Index FoP to Index FoN. **(D)** The figure shows the vaginal immune gene expression shown as ΔΔct log transformed data *C. trachomatis ompA, euo, omcB, htrA*, and *trpBA* normalized to *Ct* 16S rRNA DNA levels comparing the Index FoP to the FoP event. Data included here is sourced from biospecimens from participants in the nested case-control study (controls *n* = 22, and cases *n* = 12). The y-axis is log transformed relative expression and x-axis indicates the name of each gene of interest. Statistical significance is denoted by * (*p* ≤ 0.05), ** (*p* ≤ 0.01), and *** (*p* ≤ 0.005).

The high vaginal expression of some immune genes was analyzed in a subset of participant samples using a nested Case-Control study design (22 no-repeat infection [controls] and 12 repeat infection [cases]). The gene expression of *IRF-1* (42), *CXCL9* (43), *FTH1* (44), *IL-6* (45), *IL-8* (46), *IDO1* (47), *TNF*-α, *IL-1*α, *IL-10* (48), and *ifn*-α were determined by normalizing to the geometric mean of *GAPDH* and *PGKI* [established for the female reproductive tract (49)]. These genes were selected for the following hypothetical implications; indicative of a pro-inflammatory response needed to clear chlamydial infections (*CXCL*-9, IFN-γ, *TNF*-α, *IRF*-1, *IL*-8, *IL*-1α); biological role which may implicate host conditions compatible with chlamydial persistence (*IDO*-1, *FTH*-1); pleiotropic or reduced pro-inflammatory responses (*IL*-10 and *IL*-6) (50–52). There were no differences in the index expression of any of the selected genes between those that experienced positive or negative follow up test results. However, there was significantly lower gene expression observed at the time of follow up positive of *IL-10* and *CXCL9* when comparing to index for the same women (*p* ≤ 0.01 and ≤ 0.05, respectively) (Figure 3).

The expression of selected chlamydial genes was also analyzed from the high vaginal swabs including, the major outer membrane protein (*ompA*) (53), a repressor of genes important for late chlamydial developmental cycle stages (*euo*) (54), an outer membrane complex protein (*omcB*) (55), a protease essential for the replicative phase (*htrA*) (56), and the gene encoding the enzyme that synthesize tryptophan from indole (*trpBA*) that could be important and implicate the potential for chlamydia persistence if a host IFN-γ-IDO1 response was active in the presence of indole producing microorganisms (all normalized to 16S rRNA) (51, 57, 58). There was no difference in the expression of these genes at index comparing specimens from women experiencing FoP to FoN results, but all were found to have higher levels in women at the time of FoP compared to the same women at index (Figure 3).

## Discussion

*Chlamydia trachomatis*, as an obligate intracellular organism, has evolved over long periods of evolutionary time to adapt and survive in the cervical epithelia, a dynamic site influenced by many factors. In addition to the burden associated with testing and treating the sexually transmitted infection, *Chlamydia* is also associated with a range of serious reproductive health outcomes (2). Described here is a cohort study of chlamydial repeat infection, the aim of exploring the potential microbiological and immunological factors associated with positive tests during follow up from a treated infection in women. In this study, positive follow up test results with chlamydial vaginal infections, in women treated with azithromycin, occurred in 11.8% of women within 8 weeks of treatment. There was no evidence for chlamydial antibiotic resistance, but some indication that follow up positive infections were transcriptionally active at a higher level compared to the index (presumably longer term) infections. This higher transcriptional level of the genes tested may indicate a much more active transcription of the organism at these specific loci in response to the host-pathogen interactions in the context of repeat infections. This suggests that after treatment there may be an increased risk of repeat exposures establishing an infection, or that newly acquired infections regardless of whether repeat infections are more transcriptionally active likely due to the host not having activated an immune response.

Overall, the burden of chlamydia was higher in women who experienced positive follow up events at index by PCR, which is consistent with other studies (59, 60). In the chlamydial genomics analysis, for the 25 women where data was obtained from paired index and follow up positive eent specimens, 19 of these were clearly new infections, 3 had a small number of genotypic differences, and 3 had identical genomes.

This analysis supported four scenarios that could explain repeat infection. In the first scenario for two women in this study, genomics confirmed that the same isolate at both time points (i.e., genotypically identical) was detected (scenario 1, Table 3). In these two cases, the participant reported sexual contact (anal sex) with a repeat sexual partner who had been treated, suggesting these two cases could be accounted for by either repeat exposure, or anal auto-inoculation from an infection derived from anal sex. In the second scenario (scenario 2, Table 3), involving a single participant, the index and follow up positive isolates were genotypically identical, and this individual reported no sexual contact or no anal sex history in the time frame of the repeat infection event. This second scenario could be a treatment failure event or auto-inoculation from the anal site, having potentially acquired the anal infection *via* the hypothesized oral-anal route (13). An alternative explanation could be chlamydial persistence. A third scenario was apparent where a low number of genotypic variations was detected between the index and follow up positive sequences (24-91 SNPs, this included three participants, scenario 3, Table 3). In this third scenario two participants reported repeat sexual contact with a recent partner, and one did not report sexual contact. Given the number of genetic differences these three events could be explained by a newly acquired infection, a repeat exposure with some genetic drift over time, or anal autoinoculation with some genetic drift in host. These isolates from these three scenarios where limited genetic differences were apparent, are clearly their closest relatives, as shown in the phylogeny (Figure 2), but are not clustered together in the overall phylogeny, indicating that there is not a particular genovar or strain associated with these outcomes. The remainder of the follow up positive events from the women included in this paired genomic analysis had between 301 and 4,725 SNP differences between the index and FoP event (with repeat infections observed from 28 to 56 days, including 19 women), these cases have such a degree of genomic difference they are clearly new infections. This data also demonstrates that the genome sequencing approach may have benefits for other studies (such as future vaccine trials) where monitoring new exposure compared to clearance of organisms could be critical to monitor efficacy. This approach whilst costly presents a robust alternative to reliance on self-reported sexual behavior which is well known to have limited validity (61).

Selected chlamydial genes were highly expressed during repeat infections, compared to index. Two immune genes were observed in the gene expression analysis, *IL-10* and *CXCL9*, to be lower during the repeat infection compared to baseline. Il-10 is associated with reduced pro-inflammatory responses and immune suppression, in particular suppression of IFN-γ and other pro-inflammatory cytokine production, and in the past has been associated with reduced protection against chlamydia infection, or reduced cell mediated responses to chlamydia in murine models (48, 62–64). It was surprising to see lower levels of IL-10 in the FoP event compared to index in these women, although IFN-γ gene expression was not significantly increased relative to index in the same participants, so this reduced IL-10 gene expression may not be translating to a biological impact on the cytokine levels. The chemokine CXCLl9 has been associated with pro-inflammatory responses and immunopathology in murine models when higher levels are present or the cognate receptor is inhibited but has not been associated with clearance of the infection (65, 66). The significantly lower levels here were consistent with low (but not significant) levels of other pro-inflammatory factors during repeat infections. It has been reported that a IFN-γ producing CD4+ T cell response is associated with protection against re-infection in women (67). Although we did not detect a significant change in IFN-γ gene expression levels when comparing index with FoP specimens, we do not have subsequent gene expression data from women who were FoN (67). Whilst there were no significant differences in IFN-γ gene expression levels, there did appear to be a trend toward reduced expression of IDO-1 in the repeat infection events, which would be consistent with an overall reduction in pro-inflammatory or cell mediated response in these women with repeat infections. The gene expression analysis included some host and chlamydial genes that have been associated with biological functions or distinct expression profiles during chlamydial persistence *in vitro*. However, as the genomics analysis indicated that most follow up positive events involved new isolates or genetic drift over time indicating a related isolate, this rules out chlamydia persistence as an explanation for most cases. The host genotypes were not examined here, but it is relevant to acknowledge that the genotype of the women likely factor into repeat infection, as previously reported that HLADQB1^*^06 genotype in African American women was associated with chlamydial reinfection risk (68).

The observation of lower immune gene expression corresponding with higher chlamydia gene expression during follow up positive events should be explored further in future repeat infection studies, as this may have implications for management of women at risk of repeat infections. Three possible explanations are proposed for this observation, 1. the immune suppression from azithromycin (69) may result in higher susceptibility to repeat infection upon exposure; 2. the impact of a recent previous chlamydial infection, treatment, and any subsequent alterations of the microbiome due to varied susceptibility to the therapy used could enable a more active growth of chlamydia (and potentially could also benefit other pathogens); or 3. Incident (new) infections of *Chlamydia* may in general be more active in the transcription of these specific genes (and perhaps a selection of others), regardless of recent treatment or not (unable to explore in this dataset due to the inability to reliably ascertain the duration of any infections for women at index).

There are limitations in the study that should be considered. Firstly, the study had reduced statistical power to assess associations as the sample size was limited to only 36 cases of repeat infection in the cohort. The case-control sub-analysis was implemented to allow some level of control for hormonal contraceptives, chlamydial genovar, and age of the participants. A much larger study using a multivariate approach to control for each of these factors may provide greater insights. Much of the analysis here was contingent on molecular or biological assays from specimens which was not always possible due to low organism load, lack of chlamydia present in the specimens, contaminants or other unknown factors resulting in data not being collectable from all the swabs collected. There is an inability to explore anal infection carriage to assess the likely role of anal auto-inoculation, as anal swabs were not consistently collected from women as this was an optional component. Hypothetical factors relating to immune responses, or host factors known to drive persistence were analyzed in the gene expression analysis. When gene expression levels were observed to differ between groups this may be interpreted to indicate a cellular response impacting on gene expression, or an RNA stability change for that transcript has varied. Transcript levels do not necessarily indicate biological function that the detection of the protein would imply. Further, as only a small number of genes (and samples) analyzed it is not possible to draw conclusions with respect to these potential biological responses. Finally, as this study did not include an uninfected control group it is difficult to interpret the observations here and how these various factors may vary naturally.

Overall, these data could be interpreted to indicate that post-treatment, due to a direct impact of azithromycin or the impact of the therapy on the local tissue and microbial environment, conditions may be altered, resulting in repeat chlamydial infections having distinctive gene expression profiles. In support of these scenarios microbiome shifts have been reported after azithromycin therapy (70), and distinct impacts of azithromycin on metabolism and cytokine responses have also been recently reported (71). Alternatively, it is possible that new infections are highly transcriptionally active, regardless of whether they are repeat exposures or not, as we do not know the timeframes of the infections from the index samples. Nonetheless, repeat, or new infections being highly active in women, supports the importance of timely testing and treating of chlamydia as a public health consideration. Further study is needed to understand *in vivo* the impact of treatment, how treatment may impact risk of repeat infections, and if the reproductive health consequences may vary for these women, given the known increased occurrence of adverse outcomes associated with repeat infections (72, 73).

## Data availability statement

The datasets presented in this study can be found in online repositories. The names of the repository/repositories and accession number(s) can be found below: https://www.ebi.ac.uk/ena, PRJEB12313.

## Ethics statement

The study was reviewed and ethical approval granted by the Alfred Hospital Ethics Committee and the Southern Eastern Sydney Local Health District Human Research Ethics Committee (Southern Sector). The patients/participants provided their written informed consent to participate in this study.

## Author contributions

WH: data analysis and interpretation, study design, and manuscript drafting. AL: cultured and biological analysis of chlamydia culture and conducted the MIC analysis. BW: conducted the genomics analysis and interpretation. MT: conducted and analyzed the immunological measures. PT: data analysis and interpretation and study design. LV: study design, ethics, and conduct. SP: Chlamydia PCR genovar determination and PCR load. AM, CF, and MC: study design and conduct. RM and KW: clinical recruitment. JH: study design and conduct, epidemiological analysis, data interpretation, and participant questionnaire analysis. All authors contributed to the article and approved the submitted version.

## Funding

This study was supported by National Health and Medical Research Council Project grant number APP1023239.

## Conflict of interest

The authors declare that the research was conducted in the absence of any commercial or financial relationships that could be construed as a potential conflict of interest.

## Publisher's note

All claims expressed in this article are solely those of the authors and do not necessarily represent those of their affiliated organizations, or those of the publisher, the editors and the reviewers. Any product that may be evaluated in this article, or claim that may be made by its manufacturer, is not guaranteed or endorsed by the publisher.
