# Supplementary material for: Repeat infections with chlamydia in women may be more transcriptionally active with lower responses from some immune genes
Source: Front Public Health. 2022 Oct 10;10:1012835. doi: 10.3389/fpubh.2022.1012835 (PMC9589431; doi:10.3389/fpubh.2022.1012835)
Supplement: Supplementary file 1 [file Data_Sheet_1.docx]

**Supplementary Data Results**

Table S1: Number of participants or samples included in each study analysis

| **Study component** | **Index FoN (n)** | **Index FoP (n)** | **FoP Event (n)** |
| --- | --- | --- | --- |
| **Cohort analysis (participants)** | 235 | 36 | 36 |
| **Organism load (samples) (PCR)** | 207 | 34 | 36 |
| **Organism load (samples) (IFU)** | 235 | 36 | 31 |
| **Culture (samples)** | 26 | 19 | 8 |
| **MIC (Samples)** | 26 | 19 | 8 |
| **Genome sequence (cultured isolate) (samples)** | 3 | 8 | 4 |
| **Genome sequence (sequence capture) (samples)** | Not done | 25 (31) | 25 |
| **Gene expression (samples)** | 22 | 12 | 12 |

| **Table S2 – Participant demographic data comparing the case and control groups** | | | | | |
| --- | --- | --- | --- | --- | --- |
|  | |  | **Control** | **Case** | **Significance** |
| Count | |  | 22 | 12 |  |
| Age | | Mean (±SD) | 24.09 (2.67) | 23.50 (3.96) | 0.605^1^ |
| Condom Use | | Frequency (% within group) | 11 (50) | 5 (41.7) | 0.641770^2^ |
| Oral Contraceptive Pill Use | | Frequency (% within group) | 14 (63.6) | 8 (66.7) | 0.860^2^ |
|  | |  |  |  |  |
| Serovar | E | Frequency (%) | 12 (54.5) | 6 (50) | 0.895^2^ |
|  | F | Frequency (%) | 9 (40.9) | 5 (41.7) |  |
|  | K | Frequency (%) | 1 (4.5) | 1 (8.3) |  |
| Community State Type | I | Frequency (%) | 5 (23.8) | 7 (58.3) | 0.197^2^ |
|  | II | Frequency (%) | 9 (42.9) | 2 (16.7) |  |
|  | III | Frequency (%) | 6 (28.6) | 3 (25.0) |  |
|  | IV | Frequency (%) | 1 (4.8) | 0 (0) |  |
|  | |  |  |  |  |
| ^1^ Two-tailed t-test for equality, equal variances assumed  ^2^ Two-sided asymptotic significance by Pearson Chi-Square testing  ^3^ | | | | | |

| **Table S3. Oligonucleotide sequences used during human gene expression analysis by RT-qPCR.** | | | | | | | | |  |  |
| --- | --- | --- | --- | --- | --- | --- | --- | --- | --- | --- |
| **ID** | **Accession number** | | **Sense (5’ 🡪 3’)** | | **Antisense (5’ 🡪 3’)** | | **Amplicon Size (bp)** | |  |  |
| *GAPDH* | NM_002046.5 | | CTCTCTGCTCCTCCTGTTCG | | GACCAAATCCGTTGACTCCG | | 112 | |  |  |
| *PGK1* | NM_000291.3 | | TGGAGCTCCTGGAAGGTAAAG | | AGTTGACTTAGGGGCTGTGC | | 101 | |  |  |
| *IDO1* | NM_002164.5 | | GCAAGAACGGGACACTTTGC | | TGGGTTCACATGATCGTGGATT | | 100 | |  |  |
| *IRF1* | NM_002198.2 | | CAAATCCCGGGGCTCATCTG | | CTGCTTTGTATCGGCCTGTG | | 145 | |  |  |
| *CXCL9* | NM_002416.2 | | CAGGCTCAAAATCCAATACAGGAG | | TACTGGGGTTCCTTGCACTC | | 124 | |  |  |
| *FTH1* | NM_002032.2 | | CCAGAACTACCACCAGGACTCAG | | GGTCAAAGTAGTAAGACATGGAC | | 101 | |  |  |
| *IL-6* | NM_000600.4 | | ACCCCCAGGAGAAGATTCCAAAG | | TCACCAGGCAAGTCTCCTCATTG | | 92 | |  |  |
| *IL-8* | NM_000584.3 | | GCTCTGTGTGAAGGTGCAGTTTTG | | ACCCAGTTTTCCTTGGGGTCCAG | | 92 | |  |  |
| *IL-1a* | NM_000575.4 | | CATCCTGAATGACGCCCTCAATC | | ATCTCAGGCATCTCCTTCAGCAG | | 102 | |  |  |
| *TNF-a* | NM_000594.3 | | CTTCTGCCTGCTGCACTTTG | | CTCAGCTTGAGGGTTTGCTAC | | 90 | |  |  |
| *IL-10* | NM_000572.3 | | TACGGCGCTGTCATCGATTT | | ACTCATGGCTTTGTAGATGCCT | | 110 | |  |  |
| *IFN-g* | NM_000619.2 | | ATTGGAAAGAGGAGAGTGACAG | | CACTCTTTTGGATGCTCTGGTC | | 105 | |  |  |
|  | | **Oligonucleotide sequences used during chlamydial gene expression analysis by RT-qPCR.** | | | | | | | |  |
| **ID** | | **Sense (5’ 🡪 3’)** | | **Antisense (5’ 🡪 3’)** | | **Amplicon Size (bp)** | | **Ref** | | |
| *16S rRNA* | | GGAGAAAAGGGAATTTCACG | | TCCACATCAAGTATGCATCG | | 173 | | [296] | | |
| *omcB* | | GTAAGAGCACAAACTCCTGG | | GCACATATGAGTAGCAGCAA | | 129 | |  | | |
| *htrA* | | GACTGGGCTATTGCTATTGG | | ATGGCAGCATCTGTTTGAAT | | 131 | |  | | |
| *euo* | | TCCCCGACGCTCTCCTTTCA | | CTCGTCAGGCTATCTATGTTGCT | | 263 | | [297] | | |
| *ompA* | | TGCCGCTTTGAGTTCTGCTT | | GTCGATCATAAGGCTTGGTTCAG | | 75 | | [298] | | |
| *trpBA* | | GCATTGGAGTCTTCACATGC | | ACACCTCCTTGAATCAGAGC | | 258 | | [299] | | |
|  | |  | |  | |  | |  | | |

**Table S4. Genomic data set summary**

|  | | | |  | |  | |  | | |  | |  | | |  |  |  |
| --- | --- | --- | --- | --- | --- | --- | --- | --- | --- | --- | --- | --- | --- | --- | --- | --- | --- | --- |
| **Isolate ID** | | **Filtered Reads** | | | **Average coverage** | | | | **Accession number** | | | | | | | |  |  |
| Cultured isolates | | | | | | | | | | | | | | | |  |  |  |
| 26-049 | | 404698 | | | 114 | | | | ERR10013542 | | | | | | | |  |  |
| 26-018 | | 169353 | | | 49 | | | | ERR10013539 | | | | | | | |  |  |
| 26-010 | | 86502 | | | 24 | | | | ERR10013536 | | | | | | | |  |  |
| 26-052 (1) | | 266153 | | | 78 | | | | ERR10013543 | | | | | | | |  |  |
| 26-020 (1) | | 533071 | | | 154 | | | | ERR10013540 | | | | | | | |  |  |
| 26-028 (1) | | 586730 | | | 172 | | | | ERR10013541 | | | | | | | |  |  |
| 26-017 (1) | | 1726462 | | | 471 | | | | ERR10013537 | | | | | | | |  |  |
| 26-017 (13) | | 809405 | | | 242 | | | | ERR10013538 | | | | | | | |  |  |
| 26-114 (1) | | 1053986 | | | 52 | | | | ERR10013544 | | | | | | | |  |  |
| 26-114 (13) | | 1045634 | | | 275 | | | | ERR10013545 | | | | | | | |  |  |
| 26-131 (1) | | 1058336 | | | 193 | | | | ERR10013546 | | | | | | | |  |  |
| 26-131 (13) | | 1045719 | | | 259 | | | | ERR10013547 | | | | | | | |  |  |
| 27-554 (1) | | 1050295 | | | 171 | | | | ERR10013548 | | | | | | | |  |  |
| 27-554 (13) | | 1050428 | | | 180 | | | | ERR10013549 | | | | | | | |  |  |
| 27-610 (1) | | 1046031 | | | 200 | | | | ERR10013550 | | | | | | | |  |  |
|  | |  | | |  | | | |  | | |  | |  | | | |  |
| **SEQUENCE CAPTURE** | | | | | | | | | | | | | | |  |  |  |  |
| **Sample** | **Baseline compared to Repeat**  **Number of genomic differences** | | **Baseline**  **Average Read depth** | | **Repeat Infection**  **Average Read depth** | | **Days between initial and repeat infection** | | | **Accession number** | | | | |  |  |  |  |
| 26-017 | 0 | | 1596 | | 1461 | | 28 | | | ERR10013484, ERR10013485 | | | | |  |  |  |  |
| 26-114 | 0 | | 1499 | | 1566 | | 42 | | | ERR10013498, ERR10013499 | | | | |  |  |  |  |
| 26-124 | 0 | | 718 | | 983 | | 28 | | | ERR10013500, ERR10013501 | | | | |  |  |  |  |
| 26-131 | 24 | | 397 | | 54 | | 42 | | | ERR10013502, ERR10013503 | | | | |  |  |  |  |
| 27-586 | 31 | | 604 | | 1363 | | 42 | | | ERR10013521, ERR10013522 | | | | |  |  |  |  |
| 26-212 | 94 | | 1600 | | 247 | | 28 | | | ERR10013507, ERR10013508 | | | | |  |  |  |  |
| 27-602 | 301 | | 67 | | 15 | | 56 | | | ERR10013524, ERR10013525 | | | | |  |  |  |  |
| 26-010 | 329 | | 1006 | | 17 | | 28 | | | ERR10013480, ERR10013481 | | | | |  |  |  |  |
| 27-632 | 334 | | 1142 | | 16 | | 28 | | | ERR10013529, ERR10013530 | | | | |  |  |  |  |
| 26-233 | 421 | | 1555 | | 8 | | 42 | | | ERR10013509, ERR10013510 | | | | |  |  |  |  |
| 26-052 | 464 | | 1418 | | 369 | | 56 | | | ERR10013490, ERR10013491 | | | | |  |  |  |  |
| 26-174 | 512 | | 570 | | 27 | | 42 | | | ERR10013504, ERR10013505 | | | | |  |  |  |  |
| 26-103 | 570 | | 897 | | 1258 | | 28 | | | ERR10013496, ERR10013497 | | | | |  |  |  |  |
| 26-016 | 640 | | 1205 | | 39 | | 56 | | | ERR10013482, ERR10013483 | | | | |  |  |  |  |
| 26-079 | 681 | | 887 | | 32 | | 56 | | | ERR10013492, ERR10013493 | | | | |  |  |  |  |
| 26-237 | 735 | | 28 | | 1570 | | 56 | | | ERR10013513, ERR10013514 | | | | |  |  |  |  |
| 26-048 | 762 | | 893 | | 8 | | 42 | | | ERR10013488, ERR10013489 | | | | |  |  |  |  |
| 27-683 | 799 | | 938 | | 786 | | 56 | | | ERR10013534, ERR10013535 | | | | |  |  |  |  |
| 27-610 | 805 | | 1300 | | 1501 | | 28 | | | ERR10013526, ERR10013527 | | | | |  |  |  |  |
| 26-244 | 1267 | | 926 | | 35 | | 42 | | | ERR10013515, ERR10013516 | | | | |  |  |  |  |
| 26-235 | 1324 | | 700 | | 13 | | 56 | | | ERR10013511, ERR10013512 | | | | |  |  |  |  |
| 27-519 | 2438 | | 1256 | | 22 | | 42 | | | ERR10013519, ERR10013520 | | | | |  |  |  |  |
| 26-082 | 3506 | | 1506 | | 30 | | 56 | | | ERR10013494, ERR10013495 | | | | |  |  |  |  |
| 26-245 | 3898 | | 1157 | | 19 | | 56 | | | ERR10013517, ERR10013518 | | | | |  |  |  |  |
| 26-020 | 4725 | | 1429 | | 98 | |  | | | ERR10013486, ERR10013487 | | | | |  |  |  |  |
| 26-202 | - | |  | | 758 | | 28 | | | ERR10013506 | | | | |  |  |  |  |
| 27-595 | - | | 1198 | |  | | 28 | | | ERR10013523 | | | | |  |  |  |  |
| 27-614 | - | |  | | 42 | |  | | | ERR10013528 | | | | |  |  |  |  |
| 27-634 | - | | 951 | |  | |  | | | ERR10013531 | | | | |  |  |  |  |
| 27-667 | - | | 1605 | |  | |  | | | ERR10013532 | | | | |  |  |  |  |
| 27-675 | - | | 1499 | |  | |  | | | ERR10013533 | | | | |  |  |  |  |
